# Association of Serum and Fecal Bile Acid Patterns With Liver Fibrosis in Biopsy-Proven Nonalcoholic Fatty Liver Disease: An Observational Study

**DOI:** 10.14309/ctg.0000000000000503

**Published:** 2022-05-26

**Authors:** Yuki Kasai, Takaomi Kessoku, Kosuke Tanaka, Atsushi Yamamoto, Kota Takahashi, Takashi Kobayashi, Michihiro Iwaki, Anna Ozaki, Asako Nogami, Yasushi Honda, Yuji Ogawa, Shingo Kato, Kento Imajo, Takuma Higurashi, Kunihiro Hosono, Masato Yoneda, Haruki Usuda, Koichiro Wada, Miwa Kawanaka, Takumi Kawaguchi, Takuji Torimura, Masayoshi Kage, Hideyuki Hyogo, Hirokazu Takahashi, Yuichiro Eguchi, Shinichi Aishima, Noritoshi Kobayashi, Yoshio Sumida, Akira Honda, Shunsuke Oyamada, Satoru Shinoda, Satoru Saito, Atsushi Nakajima

**Affiliations:** 1 Department of Gastroenterology and Hepatology, Yokohama City University Graduate School of Medicine, Yokohama, Japan;; 2 Department of Palliative Medicine, Yokohama City University Hospital, Yokohama, Japan;; 3 Department of Clinical Cancer Genomics, Yokohama City University Hospital, Yokohama, Japan;; 4 Department of Pharmacology, Shimane University Faculty of Medicine, Shimane, Japan;; 5 Department of General Internal Medicine 2, Kawasaki Medical Center, Kawasaki Medical School, Okayama, Japan;; 6 Division of Gastroenterology, Department of Medicine, Kurume University School of Medicine, Kurume, Japan;; 7 Kurume University Research Center for Innovative Cancer Therapy, Kurume, Japan;; 8 Department of Gastroenterology, JA Hiroshima Kouseiren General Hospital, Hiroshima, Japan;; 9 Life Care Clinic Hiroshima, Hiroshima, Japan;; 10 Division of Metabolism and Endocrinology, Faculty of Medicine, Saga University, Saga, Japan;; 11 Liver Center, Saga University Hospital, Saga, Japan;; 12 Loco Medical General Institute, Saga, Japan;; 13 Department of Pathology and Microbiology, Faculty of Medicine, Saga University, Saga, Japan;; 14 Department of Oncology, Yokohama City University Hospital, Yokohama, Japan;; 15 Division of Hepatology and Pancreatology, Department of Internal Medicine, Aichi Medical University School of Medicine, Aichi, Japan;; 16 Division of Gastroenterology and Hepatology, Tokyo Medical University Ibaraki Medical Center, Ibaraki, Japan;; 17 Japanese Organization for Research and Treatment of Cancer (JORTC), JORTC Data Center, Tokyo, Japan;; 18 Department of Biostatistics, Yokohama City University School of Medicine.

## Abstract

**INTRODUCTION:**

No reports on both blood and fecal bile acids (BAs) in patients with nonalcoholic fatty liver disease (NAFLD) exist. We simultaneously assessed the serum and fecal BA patterns in healthy participants and those with NAFLD.

**METHODS:**

We collected stool samples from 287 participants from 5 hospitals in Japan (healthy control [HC]: n = 88; mild fibrosis: n = 104; and advanced fibrosis group: n = 95). Blood samples were collected and analyzed for serum BAs and 7α-hydroxy-4-cholesten-3-one (C4)—a surrogate marker for BA synthesis ability—from 141 patients. Concentrations of BAs, including cholic acid (CA), deoxycholic acid (DCA), chenodeoxycholic acid, ursodeoxycholic acid, and lithocholic acid (LCA), were measured using liquid chromatography-mass spectrometry.

**RESULTS:**

The total fecal BA concentration was significantly higher in the NAFLD group with worsening of fibrosis than in the HC group. Most of the fecal BAs were secondary and unconjugated. In the fecal BA fraction, CA, DCA, chenodeoxycholic acid, ursodeoxycholic acid, and LCA were significantly higher in the NAFLD than in the HC group. The total serum BA concentration was higher in the NAFLD group with worsening of fibrosis than in the HC group. In the serum BA fraction, CA, LCA, and C4 concentrations were significantly higher in the NAFLD than in the HC group.

**DISCUSSION:**

Fecal and serum BA and C4 concentrations were high in patients with NAFLD with worsening of fibrosis, suggesting involvement of abnormal BA metabolism in NAFLD with fibrosis progression. Abnormalities in BA metabolism may be a therapeutic target in NAFLD with fibrosis.

## INTRODUCTION

Nonalcoholic fatty liver disease (NAFLD) is one of the most common causes of chronic liver damage in the world (1, 2). NAFLD in individuals who consume little to no alcohol includes a wide range of liver diseases—from simple steatosis, which is generally considered to be nonprogressive, to nonalcoholic steatohepatitis (NASH), which can progress to cirrhosis and hepatocellular carcinoma (3–6). Natural history studies suggest that fibrosis progression occurs in 32%–37% of patients over a period of 3–6 years (7, 8) and up to 12% of cases progress to cirrhosis over a period of 8–10 years (9). Liver fibrosis is associated with reduced life expectancy in patients with NAFLD (10). However, the pathogenesis of NASH and its progression to fibrosis and cirrhosis remain poorly understood.

In recent years, impaired bile acid (BA) metabolism has been shown to contribute to the pathophysiology of metabolic disorders, including NAFLD (11). Furthermore, BA accumulation has been shown to induce hepatotoxicity (12). Therefore, we focused on the association between the fibrotic pathogenesis of NAFLD and BAs. In recent years, global reports have shown elevated concentrations of serum, urinary, liver, and fecal BAs in patients with NAFLD (13–18). However, the number of samples in these studies was small, few reports focused on the degree of fibrosis, and few reports were from the Orient. Therefore, the role of abnormal BA metabolism in the pathological progression of NAFLD remains uncertain. In addition, none of these reports have analyzed serum and fecal BA concentrations simultaneously. When considering BA metabolism and intestinal circulation, serum and fecal BA information in the same participant is considered crucial. The purpose of this study was to measure the serum and fecal BA concentrations in patients with NAFLD on a large scale and to investigate BA concentrations according to the degree of fibrosis in NAFLD.

## METHODS

### Study participants

This was a multicenter, cross-sectional observational study. Between May 2016 and July 2019, we evaluated a total of 287 participants, including 88 healthy controls (HCs) and 199 patients with NAFLD who underwent liver biopsy at 5 institutions (Yokohama City University Hospital, Kawasaki Medical Center, Kurume University Hospital, JA Hiroshima Kouseiren General Hospital, and Saga University Hospital). We defined NAFLD with fibrosis stage 0–2 as mild fibrosis NAFLD (mild fibrosis [MF] group) and fibrosis stage 3–4 as advanced fibrosis NAFLD (advanced fibrosis [AF] group). The patients were categorized into 3 groups: healthy control (HC) (n = 88), MF (n = 104), and AF (n = 95). (see Supplemental Digital Content 1: Inclusion criteria, http://links.lww.com/CTG/A819) presents the inclusion criteria. We performed liver biopsy for the diagnosis and staging of NASH. The histological criterion used for the diagnosis of NAFLD was the presence of macrovesicular fatty changes in the hepatocytes, with displacement of the nuclei to the edges of the cells (19). The exclusion criteria were as follows: a history of hepatic disease, such as chronic hepatitis C or concurrent active hepatitis B (seropositive for hepatitis B surface antigen); drug-induced liver injury, including that induced by amiodarone and tamoxifen; autoimmune hepatitis; primary biliary cirrhosis; sclerosing cholangitis; hemochromatosis; α1-antitrypsin deficiency; Wilson disease; hereditary disorders, including celiac disease; hepatic injury caused by substance abuse; and a current or past history of daily consumption of >20 g of alcohol.

This clinical study was conducted at 5 sites in accordance with the principles of the Declaration of Helsinki and was approved by the local ethics committees of Yokohama City University Hospital, Kawasaki Medical Center, Kurume University Hospital, JA Hiroshima Kouseiren General Hospital, and Saga University Hospital. Informed consent was obtained from all participants before enrollment. The study was registered as UMIN000020917 (University Hospital Medical Information Network).

### Patient and public involvement

Patients were involved in the conduct of the study. In particular, the development of the research question was based on the patients' experiences. The research question was explained to the representatives of the patient with NAFLD group. The results of this study will be disseminated in an international report to patients and medical staff.

### Clinical and laboratory evaluation

Blood samples were collected after 12 hours of overnight fasting. Laboratory tests were performed using the standard techniques. Blood endotoxin activity assays were performed as described previously (20, 21).

### Pathological evaluation

Liver biopsy samples were collected from all patients with NAFLD. The procedure and method of systematic evaluation are described in Supplemental Digital Content 2: Pathological evaluation and Supplemental Digital Content 3: Fibrosis stage. Item, definitions, and stage used in this study. (http://links.lww.com/CTG/A820 and http://links.lww.com/CTG/A821).

### BA and 7α-hydroxy-4-cholesten-3-one analyses

Serum and fecal BAs and serum 7α-hydroxy-4-cholesten-3-one (C4) concentrations were measured as previously described (22, 23). Briefly, a deuterium-labeled internal standard was added to 20 μL of serum or 0.2–0.4 mg of the fecal sample that was solubilized in 5% potassium hydroxide in water at 80 °C for 20 minutes. After adding 2 mL of 0.5 M potassium phosphate buffer (pH 7.4), BAs were extracted with the Bond Elut C18 cartridges (200 mg; Agilent Technologies, Santa Clara, CA) and quantified using a liquid chromatograph mass spectrometer (Shimadzu, Kyoto, Japan).

### Statistical analyses

Data are expressed as mean ± SD, unless indicated otherwise. We analyzed the data using JMP 15.0 (SAS Institute, Cary, NC). The Student *t* test was used for univariate comparisons between the groups, and the Tukey test was used for comparison among the 3 groups. Binary variables were compared using the *χ*^2^ test. All *t* tests were two-sided, with a significance level of 5% (*P* = 0.05). Since BAs have been reported to be associated with obesity and insulin resistance, these may be confounding factors (24, 25). Therefore, as a sensitivity analysis, we compared the groups in an analysis of covariance model with body mass index (BMI) and homeostatic model assessment‐insulin resistance (HOMA-IR) as covariates as a sensitivity analysis. Area under the receiver operating characteristic curves (AUROC) was used to determine diagnostic accuracy for NAFLD with advanced fibrosis. GraphPad Prism 7 (GraphPad Software, LJ) was used to create the figures, and data are shown as mean and SE in the figure.

## RESULTS

### Participant characteristics

Using a multicenter database, 199 biopsy-proven cases of NAFLD were investigated. The baseline clinical laboratory data and results of liver biopsy specimens of HCs and patients with mild and advanced fibrosis are summarized in Table 1. The body mass index, endotoxin activity, and concentrations of aspartate aminotransferase, alanine amino transferase, gamma‐glutamyl transpeptidase, total cholesterol, triglycerides, low-density lipoproteins, hyaluronic acid, and type IV collagen 7s domain were significantly higher and those of platelets, albumin, and high‐density lipoproteins were significantly lower in patients with severe fibrosis NAFLD than in HCs. The comorbidities and medications used in each group are summarized in Table 2, with significant comorbidity and medication use in the NAFLD group.

**Table 1.** Baseline characteristics of patients in the HC and NAFLD groups

	HC (n = 88)	NAFLD (MF) (n = 104)	NAFLD (AF) (n = 95)	*P*-value		
				HC vs AF	HC vs MF	MF vs AF
Demographic						
Age (yr)	60 ± 15.4	60.2 ± 11.9	58.5 ± 14.1	0.7	0.9	0.6
Sex male, n (%)	47 (53)	47 (45)	40 (42)			
BMI	21.2 ± 2.4	27.4 ± 4.2	28.6 ± 4.2	<0.0001	<0.0001	0.051
Histology						
Steatosis, n (%)						
0	88 (100)	3 (2.9)	1 (1.1)			
1	0 (0)	50 (48.1)	51 (53.7)			
2	0 (0)	38 (36.5)	29 (30.5)			
3	0 (0)	13 (12.5)	14 (14.7)			
Lobular inflammation, n (%)						
0	88 (100)	14 (13.5)	3 (3.2)			
1	0 (0)	63 (60.6)	45 (47.4)			
2	0 (0)	26 (25)	33 (34.7)			
3	0 (0)	1 (1)	14 (14.7)			
Ballooning, n (%)						
0	88 (100)	50 (48.1)	23 (24.2)			
1	0 (0)	48 (46.2)	49 (51.6)			
2	0 (0)	6 (5.8)	20 (21.1)			
3	0 (0)	0 (0)	3 (3.2)			
NAS score	0 ± 0	3.3 ± 1.44	4.2 ± 1.61	<0.0001	<0.0001	<0.0001
Blood test						
EAA	0.07 ± 0.04	0.14 ± 0.07	0.22 ± 0.08	<0.0001	<0.0001	<0.0001
Plt	25.9 ± 4.4	21.3 ± 6.1	18.8 ± 6.4	<0.0001	<0.0001	0.007
AST	21.9 ± 5.3	47.9 ± 85.5	56.5 ± 35.6	<0.0001	0.004	0.5
ALT	17.5 ± 8	51.2 ± 32.3	69.3 ± 42.5	<0.0001	<0.0001	0.0002
γ-GTP	25.5 ± 14.5	96 ± 123	66.5 ± 51	0.002	<0.0001	0.03
Alb	4.6 ± 0.38	4.4 ± 0.45	4.2 ± 0.41	<0.0001	0.001	0.02
TC	194 ± 9	208 ± 33	210 ± 39	0.002	0.005	0.95
TG	117 ± 8.4	148 ± 70.8	156 ± 79.4	0.0001	0.003	0.6
HDL-C	57.4 ± 7.7	54.5 ± 15.8	49.3 ± 12.6	<0.0001	0.3	0.01
LDL-C	113 ± 7	124 ± 28.3	129 ± 34.5	0.0003	0.015	0.4
FBG	94 ± 3.3	116 ± 24	131 ± 33	<0.0001	<0.0001	0.0001
HbA1c	5.5 ± 0.3	6.3 ± 1	6.7 ± 1.2	<0.0001	<0.0001	0.008
Type IV collagen	3.7 ± 0.37	4.9 ± 1.2	6.7 ± 1.97	<0.0001	<0.0001	<0.0001
Hyaluronic acid	25.3 ± 8.8	55 ± 62.7	114 ± 89.9	<0.0001	0.005	<0.0001
Ferritin	150 ± 85.1	235 ± 177	267 ± 189	<0.0001	0.0009	0.3
Insulin	4.1 ± 5.2	12.5 ± 7.5	20.5 ± 11.8	<0.0001	<0.0001	<0.0001
HOMA-IR	0.96 ± 1.2	3.7 ± 2.6	6.6 ± 4.7	<0.0001	<0.0001	<0.0001

Data are presented as mean ± SD, unless noted as n (%).

AF, advanced fibrosis; Alb, albumin; ALT, alanine aminotransferase; AST, aspartate aminotransferase; BMI, body mass index; EAA, endotoxin activity assay; FBG, fasting blood sugar, HbA1c, glycated hemoglobin; HC, healthy control; HDL-C, high-density lipoprotein cholesterol; HOMA‐IR, homeostatic model assessment‐insulin resistance; LDL-C, low-density lipoprotein cholesterol; MF, mild fibrosis; NAFLD, nonalcoholic fatty liver disease; NAS, NAFLD activity score; Plt, platelets; TC, total cholesterol; TG, triglycerides; γ-GTP, gamma-glutamyl transpeptidase.

**Table 2.** Comorbidities and oral medications used in each group

	HC (n = 88)	NAFLD (MF) (n = 104)	NAFLD (AF) (n = 95)	*P* value
Comorbidity				
T2DM, n (%)	0 (0)	46 (44.2)	55 (57.9)	<0.0001
HT, n (%)	0 (0)	41 (39.4)	48 (50.5)	<0.0001
DLP, n (%)	0 (0)	78 (75.0)	62 (65.2)	<0.0001
HUA, n (%)	0 (0)	10 (9.7)	6 (6.4)	0.014
Hypothyroidism, n (%)	0 (0)	1 (1)	3 (3.2)	0.17
Concomitant drugs				
Vitamin E	0 (0)	23 (22.3)	22 (23.4)	<0.0001
Ca blocker	0 (0)	18 (17.5)	21 (22.3)	<0.0001
ARB	0 (0)	28 (27.2)	30 (31.9)	<0.0001
Diuretic	0 (0)	5 (4.9)	6 (6.4)	0.07
Statin	0 (0)	43 (41.8)	45 (47.9)	<0.0001
Fibrate	0 (0)	8 (7.8)	8 (8.5)	0.02
Ezetimibe	0 (0)	18 (17.5)	6 (6.4)	<0.0001
Metformin	0 (0)	14 (13.6)	22 (23.4)	<0.0001
DPP4i	0 (0)	15 (14.6)	20 (21.3)	<0.0001
SU	0 (0)	11 (10.7)	11 (11.7)	0.005
Pio	0 (0)	9 (8.7)	2 (2.1)	0.004
SGLT2	0 (0)	2 (1.9)	20 (21.3)	<0.0001
GLP-1	0 (0)	1 (1)	4 (4.3)	0.07
Insulin	0 (0)	5 (4.9)	5 (5.3)	0.1

Data are presented as numbers (%).

AF, advanced fibrosis; ARB, angiotensin II receptor blocker; DPP4i, dipeptidyl peptidase-4 inhibitor; DLP, dyslipidemia; GLP-1, glucagon-like peptide-1; HC, healthy control; HUA, hyperuricemia; HT, hypertension; MF, mild fibrosis; NAFLD, nonalcoholic fatty liver disease; Pio, pioglitazone; SU, sulfonylurea; SGLT2, sodium-glucose cotransporter-2 inhibitor; T2DM, type 2 diabetes mellitus.

### Fecal BA profile

**Figure 1.**
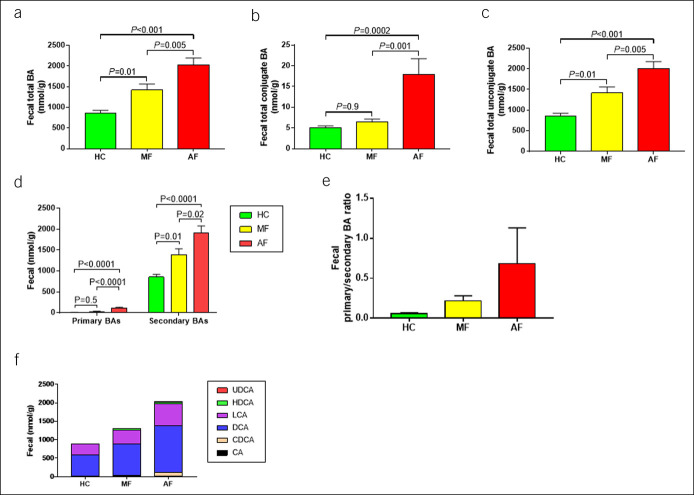
Fecal bile acid analysis among healthy controls and patients with nonalcoholic fatty liver disease with mild and advanced fibrosis. Data are presented as mean and SE. (**a**) Total BAs, (**b**) conjugated BAs, (**c**) unconjugated BAs, (**d**) primary and secondary BAs, (**e**) primary and secondary BA ratios, and (**f**) total fecal BA fraction. AF, advanced fibrosis; BA, bile acid; CA, cholic acid; CDCA, chenodeoxycholic acid; DCA, deoxycholic acid; HC, healthy control; HDCA, hyodeoxycholic acid; MF, mild fibrosis; UDCA, ursodeoxycholic acid; UDCA, ursodeoxycholic acid.

Fecal specimens were collected from all patients. The total fecal BA concentrations were higher in patients with NAFLD than in HCs, with a marked increase as fibrosis progressed (*P* < 0.0001; Figure 1a). Table 3 summarizes the distribution of fecal BAs in each group. Most were unconjugated BAs, and very few were conjugated BAs, both significantly increased as fibrosis progressed (Figure 1b,c). Most were secondary BAs, and very few were primary BAs, both significantly increased as fibrosis progressed (Figure 1d). As fibrosis progressed, the ratio of primary to secondary BAs tended to increase (Figure 1e). Regarding individual BAs, lithocholic acid (LCA) and deoxycholic acid (DCA) concentrations were high in all 3 groups (Figure 1f).

**Table 3.** Fecal bile acid profile of the HC and NAFLD groups

Bile acid (μmol/g)	HC (n = 88)	NAFLD (MF) (n = 104)	NAFLD (AF) (n = 95)	*P* value		
				HC vs AF	HC vs MF	MF vs AF
Total CA	3.0 ± 6.8	8.8 ± 14.6	22.8 ± 26.5	<0.0001	0.07	<0.0001
Unconj CA	2.4 ± 6.5	7.3 ± 13.1	18.0 ± 20.5	<0.0001	0.06	<0.0001
Conj CA	0.7 ± 1.1	1.5 ± 3.6	4.9 ± 11.9	0.0002	0.7	0.0027
Total CDCA	7.4 ± 15.2	22.2 ± 42.8	87.8 ± 205.6	<0.0001	0.7	0.0005
Unconj CDCA	7.1 ± 14.8	21.8 ± 42.3	86.2 ± 204.5	<0.0001	0.7	0.0006
Conj CDCA	0.3 ± 0.6	0.4 ± 0.7	1.6 ± 5.3	0.02	0.9	0.03
Total DCA	572.4 ± 455.0	851.8 ± 924.9	1,273.2 ± 1,332.1	<0.0001	0.12	0.008
Unconj DCA	568.9 ± 453.9	848.0 ± 922.4	1,262.7 ± 1,320.7	<0.0001	0.12	0.008
Conj DCA	3.5 ± 3.3	3.8 ± 5.0	10.4 ± 21.4	0.0009	0.99	0.0009
Total LCA	269.1 ± 229.9	503.9 ± 690.7	581.4 ± 640.4	0.0007	0.013	0.6
Unconj LCA	269.1 ± 229.9	503.8 ± 690.7	581.3 ± 640.3	0.0007	0.013	0.6
Conj LCA	0.03 ± 0.03	0.04 ± 0.04	0.05 ± 0.07	0.06	0.88	0.14
Total UDCA	2.2 ± 4.8	7.1 ± 17.6	14.5 ± 20.0	<0.0001	0.08	0.0033
Unconj UDCA	2.1 ± 4.7	7.0 ± 17.4	14.0 ± 19.3	<0.0001	0.08	0.004
Conj UDCA	0.1 ± 0.2	0.1 ± 0.3	0.5 ± 2.1	0.055	0.89	0.12
Total HDCA	9.5 ± 23.0	27.8 ± 62.7	45.3 ± 57.4	<0.0001	0.04	0.048
Unconj HDCA	9.0 ± 22.9	27.4 ± 62.7	44.7 ± 57.3	<0.0001	0.039	0.049
Conj HDCA	0.5 ± 0.3	0.5 ± 0.3	0.5 ± 0.4	0.9	0.5	0.8

Data are presented as mean ± SD.

AF, advanced fibrosis; CA, cholic acid; CDCA, chenodeoxycholic acid; Conj, conjugated; DCA, deoxycholic acid; HC, healthy control; HDCA, hyodeoxycholic acid; LCA, lithocholic acid; MF, mild fibrosis; NAFLD, nonalcoholic fatty liver disease; UDCA, ursodeoxycholic acid; Unconj, unconjugated.

Sensitivity analysis adjusted for BMI and HOMA-IR showed that fecal total BAs, especially primary BAs, were significantly increased in the advanced fibrotic NAFLD group (see **Table**, Supplemental Digital Content 4, http://links.lww.com/CTG/A822). In the fecal BA fraction, total CA (unconjugated and conjugated), total chenodeoxycholic acid (CDCA) (primarily unconjugated), and total ursodeoxycholic acid (UDCA) (unconjugated and conjugated) were significantly increased in advanced fibrotic NAFLD. However, total DCA and total LCA showed no significant differences in the advanced fibrosis NAFLD group (see **Table**, Supplemental Digital Content 4, http://links.lww.com/CTG/A822).

### Serum BA profile

**Figure 2.**
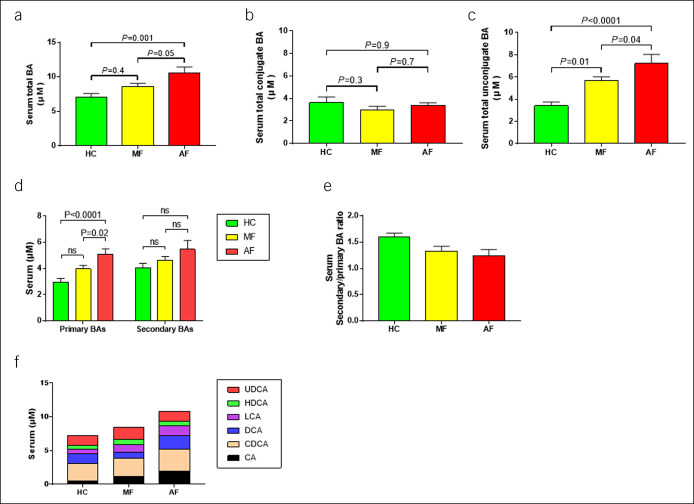
Serum bile acid analysis among healthy controls and patients with nonalcoholic fatty liver disease with mild and advanced fibrosis. Data are presented as mean and SE. (**a**) Total BAs, (**b**) conjugated BAs, (**c**) unconjugated BAs, (**d**) primary and secondary BAs, (**e**) primary and secondary BA ratios, and (**f**) total fecal BA fraction. AF, advanced fibrosis; BA, bile acid; HC, healthy control; MF, mild fibrosis.

Serum samples were collected from 141 patients (HC group: n = 55; MF group: n = 52; and AF group: n = 34). The total serum BA concentrations were higher in patients with NAFLD than in HCs, with a marked increase as fibrosis progressed (*P* = 0.001; Figure 2a). Table 4 summarizes the distribution of serum BAs in each group. There was no significant difference in conjugated BAs among the groups, whereas there were significantly more unconjugated BAs in the NAFLD group than in the HC group (more than twice the amount). Unconjugated BAs significantly increased as fibrosis progressed (Figure 2b,c). Primary BAs also showed a significant increase with the progression of fibrosis, whereas secondary BAs showed an upward tendency, but no significant difference was observed (Figure 2d). The conversion rate of primary to secondary BAs gradually decreased as fibrosis progressed (Figure 2e). Regarding individual BAs, we observed significant increases in total cholic acid (CA) and LCA between the HC and AF groups, and an increase in unconjugated CDCA between the same groups; conversely, the level of conjugated CDCA was higher in the HC than in the AF group (Figure 2f). Sensitivity analysis adjusted for BMI and HOMA-IR showed that serum total BAs, especially primary and unconjugated BAs, were significantly increased in advanced fibrotic NAFLD group (see **Table**, Supplemental Digital Content 5, http://links.lww.com/CTG/A823).

**Table 4.** Serum bile acid profile of the HC and NAFLD groups

Bile acid (μM)	HC (n = 55)	NAFLD (MF) (n = 52)	NAFLD (AF) (n = 34)	*P* value		
				HC vs AF	HC vs MF	MF vs AF
Total CA	0.44 ± 0.38	1.18 ± 0.89	1.94 ± 1.43	<0.0001	0.0002	0.0007
Unconj CA	0.27 ± 0.34	0.8 ± 0.8	1.39 ± 1.23	<0.0001	0.003	0.003
Conj CA	0.16 ± 0.08	0.38 ± 0.24	0.55 ± 0.56	<0.0001	0.0009	0.04
Total CDCA	2.59 ± 2.25	2.64 ± 1.29	3.26 ± 2.19	0.3	0.99	0.3
Unconj CDCA	1.24 ± 0.95	2.09 ± 1.3	2.45 ± 2.19	0.0006	0.008	0.5
Conj CDCA	1.36 ± 2.22	0.54 ± 0.52	0.81 ± 0.88	0.2	0.014	0.7
Total DCA	1.43 ± 1.57	0.96 ± 0.61	1.94 ± 4.76	0.6	0.6	0.2
Unconj DCA	0.45 ± 0.31	0.41 ± 0.16	1.35 ± 4.78	0.19	0.99	0.17
Conj DCA	0.98 ± 1.65	0.55 ± 0.57	0.59 ± 0.45	0.24	0.11	0.9
Total LCA	0.66 ± 0.27	1.03 ± 0.49	1.45 ± 0.71	<0.0001	0.0005	0.0005
Unconj LCA	0.46 ± 0.23	0.73 ± 0.4	1.11 ± 0.63	<0.0001	0.003	0.0002
Conj LCA	0.2 ± 0.13	0.3 ± 0.19	0.34 ± 0.22	0.0019	0.013	0.6
Total UDCA	1.4 ± 1.43	1.75 ± 1.16	1.5 ± 0.68	0.9	0.3	0.6
Unconj UDCA	0.75 ± 1.26	1.09 ± 0.73	0.83 ± 0.45	0.9	0.14	0.4
Conj UDCA	0.65 ± 0.66	0.66 ± 0.9	0.67 ± 0.38	0.9	0.9	0.9
Total HDCA	0.64 ± 0.66	0.8 ± 0.96	0.74 ± 0.47	0.8	0.5	0.9
Unconj HDCA	0.23 ± 0.32	0.33 ± 0.19	0.26 ± 0.14	0.8	0.05	0.3
Conj HDCA	0.42 ± 0.57	0.47 ± 0.98	0.48 ± 0.41	0.9	0.9	0.9

Data are presented as mean ± SD.

AF, advanced fibrosis; CA, cholic acid; CDCA, chenodeoxycholic acid; Conj, conjugated; DCA, deoxycholic acid; GCA, glycocholic acid; HC, healthy control; HDCA, hyodeoxycholic acid; LCA, lithocholic acid; MF, mild fibrosis; NAFLD, nonalcoholic fatty liver disease; UDCA, ursodeoxycholic acid; Unconj, unconjugated.

In the advanced fibrotic NAFLD group, total CA (nonconjugated and conjugated) and total LCA (mainly nonconjugated) were significantly increased in the serum BA fraction. However, total CDCA, total DCA, and total UDCA showed no such significant differences (see **Table**, Supplemental Digital Content 5, http://links.lww.com/CTG/A823).

### BA synthesis ability in the liver

**Figure 3.**
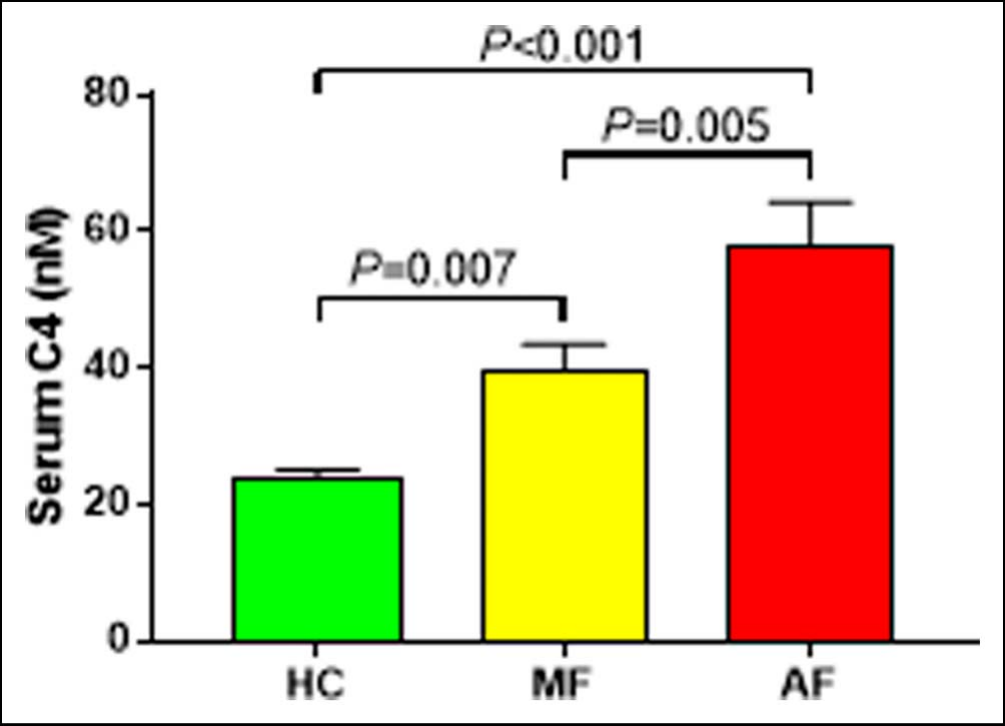
Analysis of 7α-hydroxy-4-cholesten-3-one among healthy controls and patients with nonalcoholic fatty liver disease with mild and advanced fibrosis. Data are presented as mean and SE. C4, 7α-hydroxy-4-cholesten-3-one; AF, advanced fibrosis; BA, bile acid; HC, healthy control; MF, mild fibrosis.

C4, a surrogate marker for BA synthesis, showed a significant increase as fibrosis progressed (Figure 3). The results of sensitivity analysis were similarly significantly increased with the worsening of fibrosis (see **Table**, Supplemental Digital Content 6, http://links.lww.com/CTG/A824).

### Accuracy of diagnosis for advanced fibrosis in patients with NAFLD using fecal and serum BAs and C4

BAs with an AUROC of 0.65 or greater regarding fecal BAs for the diagnosis of NAFLD advanced fibrosis were total BA (0.65), total conjugated BA (0.68), total unconjugated BA (0.65), total primary BA (0.73), total CA (0.71), total unconjugated CA (0.71), total CDCA (0.70), total unconjugated CDCA (0.70), total conjugated CDCA (0.65), total conjugated DCA (0.66), total hyodeoxycholic acid (HDCA) (0.68), total UDCA (0.69), and total unconjugated UDCA (0.69) (see **Table**, Supplemental Digital Content 7, http://links.lww.com/CTG/A825). For serum BAs, the values were total BA (0.65), total conjugated BA (0.67), total primary BA (0.66), total CA (0.67), total unconjugated CA (0.67), total DCA (0.66), total unconjugated DCA (0.66), total LCA (0.69), and total unconjugated LCA (0.71) (see **Table**, Supplemental Digital Content 7, http://links.lww.com/CTG/A825). Serum C4 was 0.67 (see **Table**, Supplemental Digital Content 7, http://links.lww.com/CTG/A825). None of the parameters had an AUROC greater than 0.8.

## DISCUSSION

**Figure 4.**
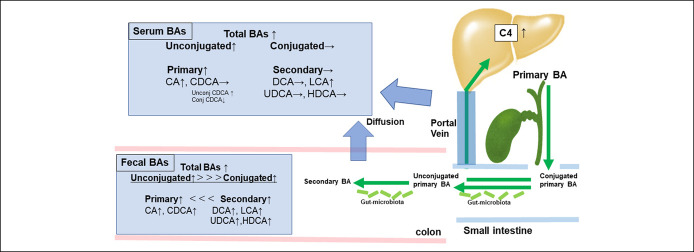
Schematic overview of the bile acid profile of patients with nonalcoholic fatty liver disease with advanced fibrosis. BA, bile acid; C4, 7a-hydroxy-4-cholesten-3-one; CA, cholic acid; CDCA, chenodeoxycholic acid; Conj, conjugated; DCA, deoxycholic acid; HDCA, hyodeoxycholic acid; LCA, lithocholic acid; UDCA, ursodeoxycholic acid; Unconj, unconjugated.

This is the first report from Asia to compare and examine the progression of NAFLD fibrosis, serum BA concentration/synthesis ability, and fecal BA concentration (Figure 4). The strength of this study is that it included a large number of biopsy-proven samples. Previous studies have reported elevated total BA concentrations in the serum, urine, liver, and feces of patients with NAFLD (13–17), although none have been conducted on such a large scale or reported simultaneously on fecal and serum samples.

BAs are important regulators of nutrient digestion and metabolism, and their homeostasis is regulated by the gut microbiota (26). Primary BAs, which are synthesized from cholesterol and bound in the liver, are excreted in the intestinal tract as digestive enzymes; reabsorbed at the terminal ileum in a process termed enterohepatic circulation; and modified in the intestine by the gut microbiota to form secondary BAs. The gut microbiota regulates the size and composition of BA pools through their effects on BA metabolism, namely, synthesis and uncoupling, and conversion of primary to secondary BAs (27, 28). Conversely, BAs are said to have the potential to change the composition of the intestinal flora by exerting antibacterial activity through the effect of surfactants on bacterial cell membranes, and they are interrelated (29). In this study, fecal BA concentrations increased with fibrosis in almost all BA fractions, with the majority being secondary and unconjugated BAs. When BAs are secreted into the intestinal tract, they are mostly primary and conjugated and are then converted to the secondary and unconjugated types by intestinal bacteria.

BAs are also known to be a major confounding factor for BMI and insulin resistance (24, 25). Analysis of fecal BAs showed that almost all BA fractions (CA, CDCA, DCA, and LCA) were increased, but when adjusted for BMI and HOMA-IR, CA and CDCA showed significant differences in the advanced fibrotic NAFLD group, while secondary BAs of DCA and LCA showed no significant differences. These results suggest that fecal DCA and LCA are affected by obesity and insulin resistance, but liver fibrosis may be an independent factor for fecal CA and CDCA.

In addition, the conversion rate of primary to secondary BAs tended to decrease as the fibrotic pathology progressed. This is consistent with the findings of a report by Mouzaki et al. (13) and may reflect changes in the gut microbiota and an increase in total BA content. Primary BAs increase intestinal permeability through autophosphorylation of epidermal growth factor receptors, dephosphorylation of occludin, and rearrangement of tight junctions (30). Secondary BAs are proinflammatory in the colon (31). Thus, patients with NAFLD are believed to have increased intestinal permeability, which is associated with metabolic endotoxinemia, insulin resistance, and the release of inflammatory cytokines, a common finding in these patients (32, 33). Our study showed that not only type IV collagen 7s and hyaluronic acid, which are typical fibrosis makers, but also blood endotoxin level increased with the worsening of liver fibrosis. Previous studies have reported that 2 intestinal factors (abnormal increase in endotoxin-producing bacteria and leakage of endotoxin into the blood due to intestinal barrier disruption) are believed to be involved in the mechanism of NAFLD progression through hyperendotoxemia (34, 35). This suggests that apart from direct hepatotoxicity, BAs may contribute to the progression of NAFLD through hyperendotoxemia by affecting intestinal permeability.

In the serum, CA, LCA, and unconjugated CDCA concentrations tended to increase and conjugated CDCA concentration tended to decrease with the progression of NAFLD. Results adjusted for BMI and HOMA-R show a similar trend, suggesting that the serum BA profile is influenced by the severity of fibrosis rather than by obesity or insulin resistance. Moreover, we suggested that the supply of BAs to the blood is related to leakage from the BA pool and diffusion from the intestinal tract; one factor is considered to be the abovementioned change in intestinal permeability. The finding regarding the decrease in conjugated CDCA concentration in this study differed from that reported by Caussy et al. (14) who reported an increase in the conjugated CDCA ratio. We speculate that changes in the gut microbiota may have caused increased conversion from conjugated to unconjugated BAs in our patient cohort. The gut microbiota has racial disparities, which may lead to varied consequences. Among the secondary BAs, only LCA showed a significant increase in serum concentration. BAs have been shown to be involved in hydrophobicity and toxicity (36) and are said to be more hydrophobic in the order of ursodeoxycholic acid <CA <CDCA <DCA <LCA (37). LCA, which is considered the most hydrophobic BA, may affect intestinal permeability and have increased diffusion into the blood. In addition, as mentioned before, the accumulation of BAs is said to be hepatotoxic (12). Increased BA concentrations, especially LCA, which is considered highly hydrophobic and toxic, may have contributed to the development of NAFLD pathology through hepatotoxicity.

In this study, the concentration of C4—which exhibits the ability to synthesize BAs—increased with fibrosis. Results adjusted for BMI and insulin resistance were also significantly increased in NAFLD patients with advanced fibrosis, suggesting that severity of liver fibrosis may be involved in the C4 increase. This is consistent with the findings of Mouzaki et al. (13), in which serum C4 concentrations increased in NAFLD and NASH. Essentially, C4 should be inhibited by an increased BA concentration. Our results indicate that the BA pool-mediated feedback mechanism may not function properly in patients with NAFLD. Other factors that suppress C4 include the fibroblast growth factor and farnesoid X receptor (38). It has been reported that the fibroblast growth factor concentration is decreased in patients with NAFLD (39–41), which may have been responsible for the increase in C4 concentration; conversely, it has also been reported that the fibroblast growth factor concentration is not decreased in NAFLD (13, 42).

We also examined the diagnosis of NAFLD fibrosis in association with BAs. Sumida et al. reported that the non-invasive liver fibrosis assessment (FIB-4) index, NAFLD fibrosis score, and aspartate aminotransferase to platelet ratio index have an AUROC greater than 0.8 (43, 44). However, as shown in Supplemental Digital Content 7 (see Table, http://links.lww.com/CTG/A825), a single fecal or serum BA, or C4, does not provide much diagnostic accuracy because AUROC does not exceed 0.8. However, it will be important in the future to examine the accuracy of combining BAs with FIB-4, NAFLD fibrosis score, and aspartate aminotransferase to platelet ratio index, for the diagnosis of advanced fibrosis in NAFLD.

Our results suggest that there is excess BA production in the pathogenesis of NAFLD, unlike the BA stasis observed in primary biliary cholangitis and viral cirrhosis. In addition, although the excess BAs were excreted in large amounts through the feces, the serum BA concentration was high, as was the BA synthesis performance, indicating that BA homeostasis could not be maintained. This new finding indicates that the pathological abnormality leading to this cycle may be important for new therapeutic approaches.

The strengths of this study are (1) a large cohort size and (2) obtaining simultaneous serum and fecal BAs. The limitations of this study include (1) the absence of gut microbiome analysis, (2) uncertainty if the association between BAs and NAFLD fibrosis is a cause or consequence, and (3) inclusion of patients from a single race (Japanese only).

In the future, by identifying the intestinal flora that is strongly involved in LCA and the conversion of primary to secondary BA (such as LCA), as well as factors related to the disruption of homeostasis in BA metabolism, it might be possible to treat NAFLD by modifying BA metabolism and intestinal bacteria.

In conclusion, the results of this study demonstrated that in patients with NAFLD, serum and fecal BA concentrations and the ability to synthesize BA increased with fibrosis. Originally, serum BA concentration should be strictly controlled; however, our findings suggest that abnormal BA metabolism may be involved in the pathogenesis of fibrosis in patients with NAFLD. Further studies are warranted to identify new therapeutic targets for the diagnosis and treatment of NAFLD by further understanding the individual factors involved in the development of NAFLD and changes in intestinal bacteria.

## CONFLICT OF INTEREST

**Guarantor of the article:** Takaomi Kessoku, MD, PhD

**Specific author contributions:** Y.K., T.K., and A.N.: contributed to the study design. Y.K., M.I., T.K., T.K., M.K., T.K., T.T., M.K., H.H., H.T., Y.E., S.S., and A.N.: responsible for data collection. Y.K., M.I., T.K., and A.H.: involved in data analysis. All authors contributed to review and writing of the manuscript. Y.K., T.K., A.H., and A.N.: responsible for the preparation of the tables and figures.

**Financial support:** The authors have not received a specific grant for this research from any funding agency in the public, commercial, or not-for-profit sectors.

**Potential competing interests:** A.N. reports grants and research support from Gilead, Mylan EPD, EA Pharma, Kowa, Taisho, and Biofermin and is a consulting adviser for Gilead, Boehringer Ingelheim, BMS, Kowa, Astellas, EA Pharma, and Mylan EPD. The remaining authors declare no competing interests.

**IRB approval statement:** This clinical study was conducted at 5 sites in accordance with the principles of the Declaration of Helsinki and was approved by the local ethics committees of Yokohama City University Hospital, Kawasaki Medical Center, Kurume University Hospital, JA Hiroshima Kouseiren General Hospital, and Saga University Hospital. Informed consent was obtained from all participants before enrollment. The study was registered as UMIN000020917 (University Hospital Medical Information Network).

**Study Highlights**

**WHAT IS KNOWN**

✓ Total serum and fecal bile acid levels are elevated in patients with nonalcoholic fatty liver disease (NAFLD).

**WHAT IS NEW HERE**

✓ This is the first report on this subject in the East.
✓ Bile acid pattern in serum and feces and the rate of synthesis were assessed simultaneously.
✓ Cholic acid and lithocholic acid were specifically elevated in the blood.
✓ Homeostasis of bile acid metabolism may not be maintained in patients with NAFLD.

## Supplementary Material
