# Supplementary material for: Association of Serum and Fecal Bile Acid Patterns With Liver Fibrosis in Biopsy-Proven Nonalcoholic Fatty Liver Disease: An Observational Study
Source: Clin Transl Gastroenterol. 2022 May 26;13(7):e00503. doi: 10.14309/ctg.0000000000000503 (PMC10476812; doi:10.14309/ctg.0000000000000503)
Supplement: Supplementary file 1 [file ct9-13-e00503-s001.docx]

**Supplemental Digital Content 1: Inclusion criteria**

We obtained the detailed history and performed ultrasonography on all NAFLD patients and control participants. Fatty liver was diagnosed based on the presence of a diffuse increase of fine echoes in the liver parenchyma, compared to the kidney or spleen parenchyma, deep beam attenuation, and bright vessel walls. The exclusion criteria were the use of antibiotics and consumption of fermented foods within 1 month of obtaining consent, cryptogenic cirrhosis, other hepatic diseases, such as hepatitis C, hepatitis B, autoimmune hepatitis, primary biliary cirrhosis, sclerosing cholangitis, Wilson’s disease, hemochromatosis, α1-antitrypsin deficiency, drug-induced steatohepatitis, or history of consumption of 30 g/day or 20 g/day of ethanol in men and women, respectively. Patients had no clinical features of hepatic decompensation such as variceal bleeding, ascites, hepatic encephalopathy, or rise of serum bilirubin level to more than twice the normal upper limit.
