# Supplementary material for: Association of Serum and Fecal Bile Acid Patterns With Liver Fibrosis in Biopsy-Proven Nonalcoholic Fatty Liver Disease: An Observational Study
Source: Clin Transl Gastroenterol. 2022 May 26;13(7):e00503. doi: 10.14309/ctg.0000000000000503 (PMC10476812; doi:10.14309/ctg.0000000000000503)
Supplement: Supplementary file 2 [file ct9-13-e00503-s002.docx]

**Supplemental Digital Content 2:** **Pathological evaluation**

Liver biopsy samples were collected from all non-alcoholic fatty liver disease (NAFLD) patients using a 16- or 18-G needle. Liver biopsy specimens were stained with hematoxylin and eosin and Masson’s trichrome stain and analyzed by two experienced pathologists (M. Kage and S. Aishima). We obtained two specimens from each patient to ensure enough sample mass for analysis and to minimize errors. An appropriate liver sample was defined as one measuring >16 mm in length and/or containing >10 portal tracts. Fatty liver was defined as >5% fat deposition in the sections. The histological activity score was evaluated as described previously.^1, 2^

1 Brunt EM, Janney CG, Di Bisceglie AM, et al. Nonalcoholic steatohepatitis: a proposal for grading and staging the histological lesions. Am J Gastroenterol 1999;94:2467-2474.

2 Kleiner DE, Brunt EM, Van Natta M, et al. Design and validation of a histological scoring system for nonalcoholic fatty liver disease. Hepatology 2005;41:1313-1321.
