# Supplementary material for: Association of Serum and Fecal Bile Acid Patterns With Liver Fibrosis in Biopsy-Proven Nonalcoholic Fatty Liver Disease: An Observational Study
Source: Clin Transl Gastroenterol. 2022 May 26;13(7):e00503. doi: 10.14309/ctg.0000000000000503 (PMC10476812; doi:10.14309/ctg.0000000000000503)
Supplement: Supplementary file 3 [file ct9-13-e00503-s003.docx]

**Supplemental Digital Content 3: Fibrosis stage. Item, definitions, and stage used in this study.**

| Item | Definition | Stage |
| --- | --- | --- |
| Fibrosis |  |  |
|  | Isolated perisinusoidal or portal/periportal fibrosis | 1 |
|  |  |  |
|  | Perisinusoidal and portal/periportal fibrosis | 2 |
|  |  |  |
|  | Bridging fibrosis | 3 |
|  |  |  |
|  | Cirrhosis | 4 |
|  |  |  |
