# Supplementary material for: Association of Serum and Fecal Bile Acid Patterns With Liver Fibrosis in Biopsy-Proven Nonalcoholic Fatty Liver Disease: An Observational Study
Source: Clin Transl Gastroenterol. 2022 May 26;13(7):e00503. doi: 10.14309/ctg.0000000000000503 (PMC10476812; doi:10.14309/ctg.0000000000000503)
Supplement: Supplementary file 4 [file ct9-13-e00503-s004.docx]

**Supplemental Digital Content 4: Sensitivity analysis of fecal bile acid profile of the HC and NAFLD groups using the analysis of covariance model with BMI and HOMA-IR as covariates.**

|  |  |  |  |  |  |  |  |  |  |  |  |  |  |  |  |  |  |  |  |  |  | P-value | | |
| --- | --- | --- | --- | --- | --- | --- | --- | --- | --- | --- | --- | --- | --- | --- | --- | --- | --- | --- | --- | --- | --- | --- | --- | --- |
|  |  |  |  |  |  |  |  |  |  |  |  |  |  |  |  |  |  |  |  |  |  | HC | HC | MF |
| Fecal bile Acid (μmol/g) | | HC | | | | | |  | NAFLD (MF) | | | | |  |  | NAFLD (AF) | | | | |  | vs | vs | vs |
|  |  | (n = 88) | | | | |  |  | (n = 104) | | | | |  |  | (n = 95) | | | | |  | AF | MF | AF |
| *Total BA* | | 1096.3 | ( | 731.4 | - | 1461.3 | ) |  | 1394.6 | ( | 1128.9 | - | 1660.4 | ) |  | 1813.8 | ( | 1493.1 | - | 2134.5 | ) | 0.03 | 0.4 | 0.1 |
|  | Conj BA | 5.3 | ( | -0.7 | - | 11.3 | ) |  | 6.3 | ( | 2.0 | - | 10.7 | ) |  | 17.9 | ( | 12.6 | - | 23.2 | ) | 0.02 | 1.0 | 0.002 |
|  | Unconj BA | 1091.0 | ( | 728.1 | - | 1453.9 | ) |  | 1388.3 | ( | 1124.1 | - | 1652.5 | ) |  | 1795.9 | ( | 1477.0 | - | 2114.7 | ) | 0.04 | 0.4 | 0.1 |
|  |  |  |  |  |  |  |  |  |  |  |  |  |  |  |  |  |  |  |  |  |  |  |  |  |
|  | Primary BA | 6.9 | ( | -28.3 | - | 42.2 | ) |  | 30.8 | ( | 5.2 | - | 56.4 | ) |  | 114.9 | ( | 84.0 | - | 145.9 | ) | 0.0003 | 0.6 | <.0001 |
|  | Secondary BA | 1089.4 | ( | 729.3 | - | 1449.5 | ) |  | 1363.8 | ( | 1101.7 | - | 1626.0 | ) |  | 1698.9 | ( | 1382.5 | - | 2015.3 | ) | 0.08 | 0.5 | 0.2 |
|  | S/P ratio | 0.2 | ( | -0.5 | - | 0.9 | ) |  | 0.2 | ( | -0.3 | - | 0.7 | ) |  | 0.5 | ( | -0.1 | - | 1.2 | ) | 0.8 | 1.0 | 0.7 |
|  |  |  |  |  |  |  |  |  |  |  |  |  |  |  |  |  |  |  |  |  |  |  |  |  |
| *Total CA* | | 3.6 | ( | -1.3 | - | 8.6 | ) |  | 8.3 | ( | 4.7 | - | 11.8 | ) |  | 23.1 | ( | 18.8 | - | 27.5 | ) | <.0001 | 0.3 | <.0001 |
|  | Unconj CA | 3.4 | ( | -0.7 | - | 7.4 | ) |  | 6.7 | ( | 3.8 | - | 9.7 | ) |  | 17.8 | ( | 14.2 | - | 21.3 | ) | <.0001 | 0.4 | <.0001 |
|  | Conj CA | 0.3 | ( | -1.7 | - | 2.3 | ) |  | 1.5 | ( | 0.1 | - | 2.9 | ) |  | 5.4 | ( | 3.6 | - | 7.1 | ) | 0.003 | 0.6 | 0.002 |
| *Total CDCA* | | 3.3 | ( | -30.2 | - | 36.8 | ) |  | 22.5 | ( | -1.8 | - | 46.9 | ) |  | 91.8 | ( | 62.4 | - | 121.3 | ) | 0.002 | 0.7 | 0.001 |
|  | Unconj CDCA | 3.2 | ( | -30.1 | - | 36.6 | ) |  | 22.1 | ( | -2.1 | - | 46.4 | ) |  | 90.1 | ( | 60.8 | - | 119.3 | ) | 0.003 | 0.7 | 0.001 |
|  | Conj CDCA | 0.1 | ( | -0.8 | - | 0.9 | ) |  | 0.4 | ( | -0.2 | - | 1.0 | ) |  | 1.7 | ( | 1.0 | - | 2.5 | ) | 0.04 | 0.8 | 0.02 |
| *Total DCA* | | 765.6 | ( | 497.2 | - | 1034.0 | ) |  | 821.2 | ( | 625.8 | - | 1016.6 | ) |  | 1126.8 | ( | 890.9 | - | 1362.7 | ) | 0.2 | 0.9 | 0.11 |
|  | Unconj DCA | 761.2 | ( | 494.4 | - | 1028.0 | ) |  | 817.4 | ( | 623.2 | - | 1011.7 | ) |  | 1117.1 | ( | 882.7 | - | 1351.5 | ) | 0.2 | 0.9 | 0.11 |
|  | Conj DCA | 4.4 | ( | 0.9 | - | 7.9 | ) |  | 3.8 | ( | 1.2 | - | 6.3 | ) |  | 9.7 | ( | 6.6 | - | 12.8 | ) | 0.1 | 1.0 | 0.01 |
| *Total LCA* | | 312.1 | ( | 159.6 | - | 464.7 | ) |  | 510.0 | ( | 398.9 | - | 621.0 | ) |  | 509.6 | ( | 375.5 | - | 643.6 | ) | 0.2 | 0.1 | 1 |
|  | Unconj LCA | 312.1 | ( | 159.6 | - | 464.7 | ) |  | 509.9 | ( | 398.9 | - | 621.0 | ) |  | 509.5 | ( | 375.5 | - | 643.6 | ) | 0.2 | 0.1 | 1 |
|  | Conj LCA | 0.0 | ( | 0.0 | - | 0.0 | ) |  | 0.0 | ( | 0.0 | - | 0.0 | ) |  | 0.0 | ( | 0.0 | - | 0.1 | ) | 0.3 | 0.9 | 0.18 |
| *Total UDCA* | | 0.9 | ( | -3.3 | - | 5.1 | ) |  | 6.8 | ( | 3.7 | - | 9.9 | ) |  | 15.5 | ( | 11.8 | - | 19.2 | ) | <.0001 | 0.1 | 0.001 |
|  | Unconj UDCA | 0.9 | ( | -3.2 | - | 5.1 | ) |  | 6.7 | ( | 3.7 | - | 9.7 | ) |  | 14.9 | ( | 11.3 | - | 18.6 | ) | <.0001 | 0.1 | 0.001 |
|  | Conj UDCA | -0.1 | ( | -0.4 | - | 0.3 | ) |  | 0.1 | ( | -0.1 | - | 0.4 | ) |  | 0.6 | ( | 0.3 | - | 0.9 | ) | 0.04 | 0.7 | 0.05 |
| *Total HDCA* | | 10.7 | ( | -3.4 | - | 24.9 | ) |  | 25.9 | ( | 15.6 | - | 36.2 | ) |  | 46.9 | ( | 34.5 | - | 59.4 | ) | 0.003 | 0.2 | 0.02 |
|  | Unconj HDCA | 10.2 | ( | -3.9 | - | 24.3 | ) |  | 25.4 | ( | 15.1 | - | 35.7 | ) |  | 46.4 | ( | 34.0 | - | 58.9 | ) | 0.003 | 0.2 | 0.02 |
|  | Conj HDCA | 0.6 | ( | 0.5 | - | 0.6 | ) |  | 0.5 | ( | 0.4 | - | 0.6 | ) |  | 0.5 | ( | 0.4 | - | 0.6 | ) | 0.7 | 0.6 | 1.0 |

Data are presented as mean ± 95% confidence interval.

AF, advanced fibrosis; BA, bile acid; BMI, body mass index; CA, cholic acid; CDCA, chenodeoxycholic acid; Conj, conjugated; DCA, deoxycholic acid; GCA, glycocholic acid; HDCA, hyodeoxycholic acid; HC, healthy control; HOMA-IR, homeostasis model assessment of insulin resistance; LCA, lithocholic acid; MF, mild fibrosis; NAFLD, nonalcoholic fatty liver disease; S/P, Secondary/primary; UDCA, ursodeoxycholic acid; Unconj, unconjugated
