# Supplementary material for: Association of Serum and Fecal Bile Acid Patterns With Liver Fibrosis in Biopsy-Proven Nonalcoholic Fatty Liver Disease: An Observational Study
Source: Clin Transl Gastroenterol. 2022 May 26;13(7):e00503. doi: 10.14309/ctg.0000000000000503 (PMC10476812; doi:10.14309/ctg.0000000000000503)
Supplement: Supplementary file 5 [file ct9-13-e00503-s005.docx]

**Supplemental Digital Content 5: Sensitivity analysis of serum bile acid profile of the HC and NAFLD groups using the analysis of covariance model with BMI and HOMA-IR as covariates**

|  |  |  |  |  |  |  |  |  |  |  |  |  |  |  |  |  |  |  |  |  |  | P-value | | |
| --- | --- | --- | --- | --- | --- | --- | --- | --- | --- | --- | --- | --- | --- | --- | --- | --- | --- | --- | --- | --- | --- | --- | --- | --- |
|  |  |  |  |  |  |  |  |  |  |  |  |  |  |  |  |  |  |  |  |  |  | HC | HC | MF |
| Serum bile Acid (μM) | | HC | | | | | |  | NAFLD (MF) | | | | |  |  | NAFLD (AF) | | | | |  | vs | vs | vs |
|  |  | (n = 55) | | | | |  |  | (n = 52) | | | | |  |  | (n = 34) | | | | |  | AF | MF | AF |
| *Total BA* | | 7.1 | ( | 5.4 | - | 8.7 | ) |  | 8.4 | ( | 7.0 | - | 9.9 | ) |  | 10.9 | ( | 9.1 | - | 12.7 | ) | 0.02 | 0.5 | 0.06 |
|  | Conj BA | 3.3 | ( | 2.1 | - | 4.4 | ) |  | 3.2 | ( | 2.3 | - | 4.2 | ) |  | 3.8 | ( | 2.6 | - | 5.0 | ) | 0.8 | 1 | 0.7 |
|  | Unconj BA | 3.8 | ( | 2.5 | - | 5.1 | ) |  | 5.2 | ( | 4.1 | - | 6.3 | ) |  | 7.1 | ( | 5.7 | - | 8.5 | ) | 0.01 | 0.3 | 0.07 |
|  |  |  |  |  |  |  |  |  |  |  |  |  |  |  |  |  |  |  |  |  |  |  |  |  |
|  | Primary BA | 3.0 | ( | 2.2 | - | 3.8 | ) |  | 3.9 | ( | 3.2 | - | 4.5 | ) |  | 5.2 | ( | 4.4 | - | 6.1 | ) | 0.003 | 0.3 | 0.02 |
|  | Secondary BA | 4.1 | ( | 2.9 | - | 5.3 | ) |  | 4.6 | ( | 3.6 | - | 5.6 | ) |  | 5.6 | ( | 4.4 | - | 6.9 | ) | 0.3 | 0.8 | 0.3 |
|  | S/P ratio | 1.6 | ( | 1.3 | - | 1.8 | ) |  | 1.4 | ( | 1.2 | - | 1.6 | ) |  | 1.2 | ( | 1.0 | - | 1.5 | ) | 0.4 | 0.6 | 0.7 |
|  |  |  |  |  |  |  |  |  |  |  |  |  |  |  |  |  |  |  |  |  |  |  |  |  |
| *Total CA* | | 0.6 | ( | 0.2 | - | 0.9 | ) |  | 1.1 | ( | 0.8 | - | 1.4 | ) |  | 1.9 | ( | 1.5 | - | 2.2 | ) | <.0001 | 0.06 | 0.002 |
|  | Unconj CA | 0.4 | ( | 0.1 | - | 0.7 | ) |  | 0.7 | ( | 0.5 | - | 1.0 | ) |  | 1.3 | ( | 1.0 | - | 1.6 | ) | 0.002 | 0.4 | 0.01 |
|  | Conj CA | 0.1 | ( | 0.0 | - | 0.2 | ) |  | 0.4 | ( | 0.3 | - | 0.5 | ) |  | 0.6 | ( | 0.5 | - | 0.7 | ) | <.0001 | 0.01 | 0.04 |
| *Total CDCA* | | 2.4 | ( | 1.7 | - | 3.1 | ) |  | 2.7 | ( | 2.1 | - | 3.3 | ) |  | 3.4 | ( | 2.6 | - | 4.1 | ) | 0.2 | 0.8 | 0.3 |
|  | Unconj CDCA | 1.2 | ( | 0.7 | - | 1.7 | ) |  | 2.1 | ( | 1.7 | - | 2.6 | ) |  | 2.5 | ( | 1.9 | - | 3.1 | ) | 0.01 | 0.05 | 0.5 |
|  | Conj CDCA | 1.2 | ( | 0.7 | - | 1.8 | ) |  | 0.6 | ( | 0.2 | - | 1.1 | ) |  | 0.9 | ( | 0.3 | - | 1.5 | ) | 0.7 | 0.3 | 0.7 |
| *Total DCA* | | 1.6 | ( | 0.7 | - | 2.5 | ) |  | 0.9 | ( | 0.1 | - | 1.6 | ) |  | 1.8 | ( | 0.8 | - | 2.8 | ) | 1.0 | 0.5 | 0.2 |
|  | Unconj DCA | 0.7 | ( | -0.1 | - | 1.6 | ) |  | 0.2 | ( | -0.5 | - | 1.0 | ) |  | 1.1 | ( | 0.2 | - | 2.0 | ) | 0.8 | 0.7 | 0.2 |
|  | Conj DCA | 0.9 | ( | 0.5 | - | 1.3 | ) |  | 0.6 | ( | 0.3 | - | 0.9 | ) |  | 0.7 | ( | 0.2 | - | 1.1 | ) | 0.8 | 0.6 | 1.0 |
| *Total LCA* | | 0.8 | ( | 0.6 | - | 0.9 | ) |  | 1.0 | ( | 0.8 | - | 1.1 | ) |  | 1.4 | ( | 1.2 | - | 1.5 | ) | 0.0003 | 0.3 | 0.002 |
|  | Unconj LCA | 0.6 | ( | 0.4 | - | 0.7 | ) |  | 0.7 | ( | 0.5 | - | 0.8 | ) |  | 1.0 | ( | 0.9 | - | 1.2 | ) | 0.0005 | 0.6 | 0.0004 |
|  | Conj LCA | 0.2 | ( | 0.1 | - | 0.3 | ) |  | 0.3 | ( | 0.2 | - | 0.4 | ) |  | 0.3 | ( | 0.2 | - | 0.4 | ) | 0.1 | 0.1 | 0.9 |
| *Total UDCA* | | 1.2 | ( | 0.8 | - | 1.7 | ) |  | 1.9 | ( | 1.5 | - | 2.2 | ) |  | 1.6 | ( | 1.2 | - | 2.1 | ) | 0.5 | 0.1 | 0.7 |
|  | Unconj UDCA | 0.7 | ( | 0.4 | - | 1.0 | ) |  | 1.1 | ( | 0.8 | - | 1.4 | ) |  | 0.9 | ( | 0.5 | - | 1.2 | ) | 0.9 | 0.2 | 0.4 |
|  | Conj UDCA | 0.5 | ( | 0.3 | - | 0.8 | ) |  | 0.7 | ( | 0.5 | - | 0.9 | ) |  | 0.8 | ( | 0.5 | - | 1.0 | ) | 0.5 | 0.5 | 1.0 |
| *Total HDCA* | | 0.5 | ( | 0.2 | - | 0.7 | ) |  | 0.9 | ( | 0.7 | - | 1.1 | ) |  | 0.9 | ( | 0.6 | - | 1.2 | ) | 0.2 | 0.09 | 1.0 |
|  | Unconj HDCA | 0.2 | ( | 0.1 | - | 0.3 | ) |  | 0.3 | ( | 0.3 | - | 0.4 | ) |  | 0.3 | ( | 0.2 | - | 0.4 | ) | 0.8 | 0.1 | 0.4 |
|  | Conj HDCA | 0.3 | ( | 0.0 | - | 0.5 | ) |  | 0.6 | ( | 0.3 | - | 0.8 | ) |  | 0.6 | ( | 0.3 | - | 0.9 | ) | 0.3 | 0.3 | 1.0 |

Data are presented as mean ± 95% Confidence Interval.

AF, advanced fibrosis; BA, bile acid; BMI, body mass index; C4, 7α-hydroxy-4-cholesten-3-one; CA, cholic acid; CDCA, chenodeoxycholic acid; Conj, conjugated; DCA, deoxycholic acid; GCA, glycocholic acid; HC, healthy control; HDCA, hyodeoxycholic acid; HOMA-IR, homeostasis model assessment of insulin resistance; LCA, lithocholic acid; MF, mild fibrosis; NAFLD, nonalcoholic fatty liver disease; S/P, Secondary/primary; UDCA, ursodeoxycholic acid; Unconj, unconjugated
