# Supplementary material for: Association of Serum and Fecal Bile Acid Patterns With Liver Fibrosis in Biopsy-Proven Nonalcoholic Fatty Liver Disease: An Observational Study
Source: Clin Transl Gastroenterol. 2022 May 26;13(7):e00503. doi: 10.14309/ctg.0000000000000503 (PMC10476812; doi:10.14309/ctg.0000000000000503)
Supplement: Supplementary file 6 [file ct9-13-e00503-s006.docx]

**Supplemental Digital Content 6: Sensitivity analysis of serum C4 level of the HC and NAFLD groups using the analysis of covariance model with BMI and HOMA-IR as covariates**

|  |  |  |  |  |  |  |  |  |  |  |  |  |  |  |  |  |  |  |  |  | P-value | | |
| --- | --- | --- | --- | --- | --- | --- | --- | --- | --- | --- | --- | --- | --- | --- | --- | --- | --- | --- | --- | --- | --- | --- | --- |
|  |  |  |  |  |  |  |  |  |  |  |  |  |  |  |  |  |  |  |  |  | HC | HC | MF |
| Serum C4 (μM) | HC | | | | | |  | NAFLD (MF) | | | | |  |  | NAFLD (AF) | | | | |  | vs | vs | vs |
|  | (n = 55) | | | | |  |  | (n = 52) | | | | |  |  | (n = 34) | | | | |  | AF | MF | AF |
| C4 | 1.5 | ( | 0.8 | - | 2.2 | ) |  | 2.9 | ( | 2.3 | - | 3.5 | ) |  | 3.8 | ( | 3.1 | - | 4.6 | ) | 0.0003 | 0.02 | 0.08 |

Data are presented as mean ± 95% Confidence Interval.

AF, advanced fibrosis; BA, bile acid; BMI, body mass index; C4, 7α-hydroxy-4-cholesten-3-one; HC, healthy control; HOMA-IR, homeostasis model assessment of insulin resistance; MF, mild fibrosis; NAFLD, nonalcoholic fatty liver disease.
