# Supplementary material for: Association of Serum and Fecal Bile Acid Patterns With Liver Fibrosis in Biopsy-Proven Nonalcoholic Fatty Liver Disease: An Observational Study
Source: Clin Transl Gastroenterol. 2022 May 26;13(7):e00503. doi: 10.14309/ctg.0000000000000503 (PMC10476812; doi:10.14309/ctg.0000000000000503)
Supplement: Supplementary file 7 [file ct9-13-e00503-s007.docx]

**Supplemental Digital Content 7: Ability to diagnose advanced fibrosis in NAFLD patients using fecal and serum bile acids and C4**

| Feces | | Cut-off (μmol/g) | AUROC | Sensitivity | Specificity |  | Serum | | Cut-off (μM) | AUROC | Sensitivity | Specificity |
| --- | --- | --- | --- | --- | --- | --- | --- | --- | --- | --- | --- | --- |
| *Total BA* | | 1274.4 | 0.65 | 67 | 63 |  | *Total BA* | | 7.9 | 0.65 | 74 | 71 |
|  | Conj BA | 6.7 | 0.68 | 56 | 67 |  |  | Conj BA | 3.0 | 0.67 | 56 | 67 |
|  | Unconj BA | 1272.2 | 0.65 | 67 | 63 |  |  | Unconj BA |  | 0.62 |  |  |
|  |  |  |  |  |  |  |  |  |  |  |  |  |
|  | Primary | 12.5 | 0.73 | 74 | 60 |  |  | Primary | 4.2 | 0.66 | 64 | 70 |
|  | Secondary |  | 0.61 |  |  |  |  | Secondary |  | 0.62 |  |  |
|  | S/P ratio |  | 0.59 |  |  |  |  | S/P ratio |  | 0.60 |  |  |
|  |  |  |  |  |  |  |  |  |  |  |  |  |
| *Total CA* | | 3.2 | 0.71 | 77 | 58 |  | *Total CA* | | 1.0 | 0.67 | 71 | 64 |
|  | Unconj CA | 2.5 | 0.71 | 76 | 55 |  |  | Unconj CA | 0.8 | 0.67 | 62 | 67 |
|  | Conj CA |  | 0.64 |  |  |  |  | Conj CA |  | 0.59 |  |  |
| *Total CDCA* | | 13.0 | 0.70 | 65 | 66 |  | *Total CDCA* | |  | 0.57 |  |  |
|  | Unconj CDCA | 14.0 | 0.70 | 62 | 67 |  |  | Unconj CDCA |  | 0.53 |  |  |
|  | Conj CDCA | 0.4 | 0.65 | 44 | 76 |  |  | Conj CDCA |  | 0.60 |  |  |
| *Total DCA* | |  | 0.63 |  |  |  | *Total DCA* | | 1.0 | 0.66 | 59 | 68 |
|  | Unconj DCA | | 0.62 |  |  |  |  | Unconj DCA | 0.5 | 0.66 | 62 | 71 |
|  | Conj DCA | 2.7 | 0.66 | 66 | 70 |  |  | Conj DCA |  | 0.60 |  |  |
| *Total LCA* | |  | 0.56 |  |  |  | *Total LCA* | | 1.3 | 0.69 | 59 | 58 |
|  | Unconj LCA | | 0.56 |  |  |  |  | Unconj LCA | 1.0 | 0.71 | 47 | 64 |
|  | Conj LCA |  | 0.60 |  |  |  |  | Conj LCA |  | 0.55 |  |  |
| *Total HDCA* | | 14.0 | 0.68 | 60 | 68 |  | *Total HDCA* | |  | 0.51 |  |  |
|  | Unconj HDCA | 7.2 | 0.68 | 74 | 68 |  |  | Unconj HDCA |  | 0.62 |  |  |
|  | Conj HDCA | | 0.45 |  |  |  |  | Conj HDCA |  | 0.60 |  |  |
| *Total UDCA* | | 6.1 | 0.69 | 53 | 71 |  | *Total UDCA* | |  | 0.56 |  |  |
|  | Unconj UDCA | 6.1 | 0.69 | 53 | 61 |  |  | Unconj UDCA |  | 0.61 |  |  |
|  | Conj UDCA | | 0.59 |  |  |  |  | Conj UDCA |  | 0.62 |  |  |
|  |  |  |  |  |  |  |  |  |  |  |  |  |
|  |  |  |  |  |  |  | *C4* | | 2.4 | 0.67 | 73 | 61 |

AUROC, Area under the Receiver Operatorating Characteristic Curve; C4, 7α-hydroxy-4-cholesten-3-one; CA, cholic acid; CDCA, chenodeoxycholic acid; Conj, conjugated; DCA, deoxycholic acid; GCA, glycocholic acid; HC, healthy control; HDCA, hyodeoxycholic acid; LCA, lithocholic acid; NAFLD, nonalcoholic fatty liver disease; S/P, secondary/primary; UDCA, ursodeoxycholic acid; Unconj, unconjugated
